# Optimization of Protein Extraction from Rapeseed Oil Cake by Dephenolization Process for Scale-Up Application Using Artificial Neural Networks

**DOI:** 10.3390/foods14101762

**Published:** 2025-05-07

**Authors:** Branislava Đermanović, Jelena Vujetić, Tea Sedlar, Danka Dragojlović, Ljiljana Popović, Predrag Kojić, Pavle Jovanov, Bojana Šarić

**Affiliations:** 1 Faculty of Technology Novi Sad, University of Novi Sad, Bulevar Cara Lazara 1, 21000 Novi Sad, Serbia; branislava.djermanovic@fins.uns.ac.rs (B.Đ.); ljiljana04@tf.uns.ac.rs (L.P.); kojicpredrag@uns.ac.rs (P.K.); 2 Institute of Food Technology in Novi Sad, University of Novi Sad, Bulevar Cara Lazara 1, 21000 Novi Sad, Serbia; jelena.vujetic@fins.uns.ac.rs (J.V.); tea.sedlar@fins.uns.ac.rs (T.S.); danka.dragojlovic@fins.uns.ac.rs (D.D.); pavle.jovanov@fins.uns.ac.rs (P.J.)

**Keywords:** rapeseed cake, rapeseed protein isolates, artificial neural networks (ANNs), plant protein production, in vitro digestion

## Abstract

Rapeseed proteins, due to their quality and wide availability, have great potential for application in human nutrition. However, their high content of antinutritional compounds poses significant economic and environmental challenges for food industry applications. To overcome these obstacles, various extraction and modification techniques, including enzymatic and ultrasound-assisted methods, were used to enhance protein functionality and improve both nutritional and sensory properties. In this study, the effects of dephenolization on the structural, physicochemical, and functional properties of rapeseed protein isolate obtained from defatted rapeseed cake were investigated through four different protocols. All obtained protein isolates (PIs) exhibited high protein purity (above 65%), with a notable difference in extraction yield. Furthermore, the extraction process was optimized using an artificial neural network (ANN) model, which demonstrated high predictive performance. The optimal extraction conditions for the dephenolization of rapeseed oil cake were 84% ethanol concentration, a solid-to-liquid ratio of 1/60 *w*/*v*, and 15 min of ultrasound treatment, resulting in an impressive protein purity of 90.68% with a yield of 29.17%. The obtained proteins were further characterized and compared in terms of protein profile (FTIR and SDS-PAGE), amino acid composition, solubility, and digestibility. The protein isolate (PI) obtained under optimized conditions displayed superior functional properties, underscoring the relevance and necessity of a data-driven, mathematical approach for scale-up and industrial implementation.

## 1. Introduction

The world is facing significant challenges due to population growth, increasing demand for food, and limited energy resources. As a result, there is a growing shift toward food production based on alternative sources, better utilization, and the circular economy, in alignment with the UN Agenda 2030 [1]. The production of meat as a nutritionally rich raw material is becoming increasingly unsustainable due to harmful gas emissions, excessive water usage, and energy inefficiency [2]. Consequently, alternative protein sources and the valorization of by-products from the food industry are gaining more attention.

Rapeseed (*Brassica napus* L.) is one of the most widespread oil crops in the world. After years of continuous production growth, rapeseed reached second place in the world market with 90 million tons in 2023, and production is expected to continue increasing [3]. Alongside the oil, a valuable by-product known as rapeseed cake (RSC) is generated, which plays a significant role, mainly, in the animal feed industry. RSC contains 38–46% d.w. protein, depending on seed quality and extraction methods [4]. Scientific studies have confirmed that these are proteins of exceptional quality, with a well-balanced ratio of amino acids and a high proportion of lysine (about 6%), one of the amino acids that present limiting amino acid in cereals. All of this has led to numerous scientific projects and publications focused on isolating rapeseed protein for potential applications in the food industry.

The main protein fraction in rapeseed proteins is storage seed proteins, cruciferin and napin, which account for 85–90% of the total protein. Small amounts of structural proteins and metabolic proteins are also present. Cruciferin, an 11S globulin heteromer of 300–350 kDa, is the dominant seed storage protein. Napin, a 2S albumin of 14–16 kDa, is present in smaller amounts compared to cruciferin. These proteins contribute to excellent functional properties such as emulsification, foaming, and gelling, giving them great potential for application in food products like dressings, whipped creams, sauces, and meringues [5,6,7].

However, the main challenge for utilizing rapeseed cake as human food is the high content of antinutritional compounds, especially phenolics—tannins and sinapine [8] Their presence most significantly impacts the nutritional value and sensory acceptance of these proteins, leading to phenol–protein interactions that result in a dark color, bitter taste [5,8,9], limited solubility, potential allergenicity [10,11], and reduced protein digestibility [12].

To overcome these limitations, various pretreatment methods, such as ethanol washing (dephenolization) [13,14] and enzymatic treatments [15], have been explored to improve protein isolation and quality. These include ethanol washing (dephenolization) to reduce antinutritional factors, enzymatic treatments (cellulases, proteases, and pectinases) to enhance extraction efficiency, and advanced techniques such as ultrasonic and microwave methods to increase protein yield [16]. Additionally, ultrafiltration has been employed as an effective method for protein purification and concentration while preserving functional properties [17,18]. Given these challenges, the dephenolization of rapeseed cake is essential for obtaining protein products that are acceptable and competitive in the alternative protein market. In order to meet the requirements of large-scale industrial production for obtaining proteins of high yield and purity, it is necessary to optimize the entire process of protein isolation, which would make it comparatively more environmentally and economically sustainable.

The dephenolization process may be affected by various factors, such as solid/cake to solvent ratio, residence time, and type and concentration of solvent. When many factors and interactions affect the desired response, data-driven models such as artificial neural networks (ANNs) could be an effective tool for finding their optimal values [19,20]. Mimicking the neuronal connection in the human brain, the ANN demonstrates remarkable capabilities in learning, estimation, association, and adaptability. It requires training and fine-tuning of input parameters to enhance the accuracy of the input–output relationship. Hence, the ANN has become the trusted mathematical tool in extraction processes due to its great accuracy in mapping inputs with outputs. The use of global optimization is brought about by this analysis to find the best operating parameters to achieve the highest yield of polyphenolic. Moreover, recent studies have indicated that the ANN can effectively perform with less experimental data and that an effective ANN model can be constructed using the same components as the RSM dataset [21].

The industrial application of protein extraction methods from rapeseed processing by-products faces challenges related to economic feasibility and large-scale process optimization. Although many techniques have been successfully demonstrated under laboratory conditions, their implementation on a pilot scale and in industrial settings requires further research. To the best of our knowledge, no study has yet been conducted on the optimization of the dephenolization process using artificial neural networks (ANNs). Therefore, this study investigates the impact of various treatments and pretreatments on protein isolation from rapeseed cake, employing the ANN to optimize the removal of compounds that negatively affect the quality of the isolated proteins. The obtained proteins were characterized using techniques such as sodium dodecyl-sulfate–polyacrylamide gel Electrophoresis (SDS-PAGE), Fourier-transform infrared spectrum (FTIR), amino acid composition analysis, and in vitro protein digestibility analysis. The results of this research provide optimized strategies for pilot-scale applications, paving the way for potential industrial implementation.

## 2. Materials and Methods

### 2.1. Materials and Reagents

Cold-pressed rapeseed (*Brassica napus* L.) cake was obtained from local oil industry (Granum, Hajdukovo, Serbia) as raw material and stored at 4 °C until use. Commercially available liquid enzyme preparations were used in the experiments: Viscozyme^®^L, a multi-enzyme complex containing arbanase, β-glucanase, cellulase, hemicellulose, and xylanase, obtained from *Aspergillus aculeatus* (declared activity of 100 FBG/g) obtained from Sigma Chemical Company (Sigma Aldrich, St. Louis, MO, USA). All reagents and chemicals used in the experimental work were of analytical grade or higher. Folin–Ciocalteu reagent, Dalton Mark VII, Coomassie Brilliant Blue G-250, α-amylase, pepsin, pancreatin and Amino Acid Standard Solution were purchased from Sigma-Aldrich GmbH (Sternheim, Germany), while HCl, NaOH, and CaCl_2_ were obtained from Merck (Darmstadt, Germany) and ethanol was obtained from Zorka Pharma-Hemija (Šabac, Serbia).

Cold-pressed RSC was defatted twice using hexane extraction (1:5 solid–liquid ratio, 1000 rpm, 1 h, 25 °C), with a 30 min interval between extractions. The defatted RSC was then separated and dried overnight at 25 °C. The dried material was ground into a fine powder using a laboratory mill (KN 295, FOSS, Hilleroed, Denmark). The approximate composition of the defatted RSC was determined using the methods of the Association of Official Analytical Chemists [22] and was as follows: 40.42% protein, 1.25% oil, 6.58% ash, and 51.75% carbohydrates expressed on a dry matter basis.

### 2.2. Extraction of Proteins

#### 2.2.1. pH Shift Alkaline Extraction–Isoelectric Precipitation Protocol

Defatted RSC was mixed with deionized water at a solid-to-liquid ratio of 1:10 (*w*/*v*). The pH of the suspension was adjusted to 9.0, 10.0, 11.0, or 12.0 using 1 M NaOH, and the mixture was stirred for 60 min at room temperature on a magnetic stirrer (C-MAG HS 4, IKA, Staufen, Germany). After extraction, the slurry was filtered to separate the insoluble material, and the obtained extract underwent isoelectric precipitation at pH 4.5 by the addition of 1 M HCl. The precipitated proteins were recovered by centrifugation (Eppendorf 5804R, Hamburg, Germany) at 10,000 rpm for 20 min at 4 °C. The resulting precipitate was dried in vacuum dryer (Binder VD 115, Tuttlingen, Germany) at 40 °C for 24 h and finely ground into a powder. The optimal extraction pH was determined based on protein content and yield as predefined evaluation criteria. After initial testing, the solid-to-liquid ratio of 1:20 (*w*/*v*) was found to yield more favorable results compared to the 1:10 (*w*/*v*) ratio. Therefore, further investigations were carried out using this ratio, choosing pH 12 and testing the 1:20 (*w*/*v*) ratio.

#### 2.2.2. Pretreatments of the Selected Alkaline Extraction–Isoelectric Precipitation Protocol

##### Dephenolization

Dephenolization was performed to remove phenolic compounds that could interfere with protein isolation and reduce the efficiency of extraction. Defatted RSC was treated with 80% ethanol at a solid-to-liquid ratio of 1:20 (*w*/*v*) and mixed at 1000 rpm for 30 min at 25 °C using a magnetic stirrer (C-MAG HS 4, IKA, Staufen, Germany). After phenolic extraction, the suspension was filtered, and the obtained residue underwent the alkaline extraction protocol described in Section 2.2.1, using pH 12.0. The obtained protein isolate was dried and named Pdp.

##### Enzyme-Assisted Extraction

Enzymatic pretreatment was applied to degrade the cell wall matrix, thereby enhancing protein accessibility and release. The process was carried out using the cellulolytic complex Viscozyme^®^ L at 2.5% (*w*/*w*), relative to the dry weight of the substrate. The required volume of liquid enzyme was determined based on its density. The reaction conditions were set at pH 4.5, a temperature of 40 °C, and a solid-to-liquid ratio of 1:20. The water used for preparing the suspension was pre-adjusted to pH 4.5 prior to mixing with the sample, and the pH was confirmed and adjusted, if necessary, immediately after mixing. Mixing was provided using a propeller mixer to ensure optimal enzyme activity. Following enzymatic treatment, protein extraction was performed according to the alkaline extraction protocol described in Section 2.2.1. The sample was dried and designated as Pe.

##### Ultrasound-Assisted Extraction

To solubilize proteins, the defatted RSC was treated in alkaline conditions (pH 12), and ultrasonic treatment was subsequently applied to improve extraction efficiency. After the cake was mixed with deionized water at a solid-to-liquid ratio of 1:20 (*w*/*v*), the pH of the mixture was adjusted to 12.0 using 1 M NaOH, and the mixture was stirred for 60 min. The mixture was then subjected to ultrasonic treatment in a multi-frequency ultrasonic bath (TI-H Multi Frequencies Ultrasonic Bath, Elma Schmidbauer GmbH, Singen, Germany) at a frequency of 45 kHz and 100% power for 30 min at room temperature. Periodic manual stirring with a glass rod during ultrasonic treatment ensured uniform dispersion of ultrasonic energy throughout the mixture. After ultrasonic treatment, the suspension was filtered to remove insoluble material, and protein isolation was completed according to the method described in Section 2.2.1. The sample was dried and designated as Pus.

##### Dephenolization with Ultrasound

This protocol combines dephenolization (as described in the dephenolization section) with ultrasound-assisted extraction (as described in the ultrasound-assisted extraction section). In the initial step, the defatted rapeseed cake (RSC) sample was treated with 80% ethanol at a solid-to-liquid ratio of 1:20 (*w*/*v*), and the mixture was stirred for 30 min using a magnetic stirrer (C-MAG HS 4, IKA, Staufen, Germany) at 1000 rpm to remove phenolic compounds. The suspension was then filtered to separate the solid residue, which was subsequently used for protein extraction.

The remaining cake was suspended in deionized water at a solid-to-liquid ratio of 1:20 (*w*/*v*), and the pH of the mixture was adjusted to 12.0 using 1 M NaOH. The mixture was stirred for 60 min, followed by ultrasonic treatment in a multi-frequency ultrasonic bath (TI-H Multi Frequencies Ultrasonic Bath, Elma Schmidbauer GmbH, Singen, Germany) at a frequency of 45 kHz and 100% power for 30 min at room temperature. During sonication, the mixture was periodically stirred manually with a glass rod to ensure even distribution of ultrasonic energy throughout the sample.

After the ultrasonic treatment, the suspension was filtered to remove insoluble material, and the protein isolation procedure was continued as described in Section 2.2.1. The final sample was dried and designated as Pdp-us.

### 2.3. Determination of Protein Content and Calculation of Yield

The total protein content was analyzed using the Kjeldahl method (official AOAC 979.20) [22]. All results are expressed on a dry matter basis. The extraction yield of protein (%) was calculated using Equations (1)–(3):

(1) $$m_{1}=\frac{m_{0}\times m_{p}\;}{100}$$

*m*_0_—initial mass of RSC (g); *m_p_*—protein content of RSC.

Mass of PI after extraction, *m*_3_ (g):

(2) $$m_{3}=\frac{m_{2}\;\times \;P(\%)}{100}$$

*m*_2_—mass of dried PIs after protein extraction (g); *P* (%)—protein content in dried PIs analyzed by Kjeldahl method.

Extraction yield of protein (%):

(3) $$Yield=\frac{m_{3}\times 100}{m_{1}}$$

### 2.4. Optimization of Method for Dephenolization of RSC

#### 2.4.1. Artificial Neural Network

The dephenolization process was measured and optimized by monitoring the effects of three extraction process factors, solid to liquid phase ratio (S/L) (1/10–1/60 *w*/*v*), ultrasounds time (t) (5–25 min), and ethanol concentration (70–90%), on the extraction yield of polyphenolics. The ranges of these factors were determined based on preliminary experimental trials with RSC and literature data [19,20,23]. In subsequent experimental steps, the number of experiments was increased with the aim of maximizing the extraction yield of polyphenolic compounds and reaching a critical concentration where the extraction process reaches equilibrium. The experimental data utilized for the analysis were derived to achieve a sample size of 33, which was deemed sufficient for determining the architecture of the ANN and preventing overfitting. The presented ANN methodology elucidates the effects of the input process variables on the observed responses (polyphenol yield), identifies interrelationships between inputs, and represents the combined effect of all inputs on the observed responses, thereby enabling the comprehension of complex processes by the experimenter.

In this study, the prediction of polyphenol yield for different process parameters was conducted using ANN. To improve the quality of the ANN, both input and output data were standardized. A multilayer perceptron model consisting of three layers (input, hidden, and output) with the hyperbolic tangent function as the activation function was employed. Better results were obtained using the Levenberg–Marquardt calculation compared to other training algorithms (such as Bayesian regularization). The ANN results, including weight values, are dependent on the initial assumptions of parameters necessary for ANN construction and fitting. Similarly, varying the number of hidden neurons can lead to diverse outcomes of the ANN model. To mitigate this issue, each topology was executed multiple times to minimize random correlation resulting from initial assumptions and random weight initialization. Subsequently, the proposed ANN was utilized to determine the significance of the input parameters regarding outputs. Therefore, following the successful construction of the ANN and obtaining weight matrices, Yoon’s equation was chosen to ascertain the relative importance (RI) of the input process values and their effect on HTC yield improvement. This equation is based on the connection weights obtained from the developed ANN, as per the equation formulated by [24]:

(4) $${RI}_{ij}\left(\%\right)=\;\frac{\sum_{k=0}^{n}\left(w_{ik}w_{kj}\right)}{\sum_{i=0}^{m}abs\;\sum_{k=0}^{n}\left(w_{ik}w_{kj}\right)}\;\times \;100$$

where *RI_ij_* is the relative importance of the input variable (i) on the output (j), *w_ik_* is the weight between the input and the hidden neuron (k), and *w_kj_* is the weight between the hidden neuron and the output.

#### 2.4.2. Particle Swarm Optimization

The local minimum trap is a potential issue with standard optimization methods, such as the aforementioned response surface methodology. This led to the utilization of global search solvers, such as the particle swarm algorithm, which was integrated into the ANN architecture [25]. The particle swarm algorithm operates as a population-based method where a group of particles iteratively moves towards the converging region. Each particle’s objective function is evaluated at every step, and the algorithm calculates the new velocity of each particle accordingly. As particles continue to move, the algorithm reevaluates their positions. The optimization process was performed using MATLAB 2023b (The MathWorks, Inc., Natick, MA, USA) with the built-in function “particleswarm”. This algorithm reduces computing time compared to other methods like genetic algorithms and direct search. Furthermore, variations in populations and hybrid functions were explored, yet the solution’s quality remained consistent.

#### 2.4.3. Alkaline Extraction of Proteins from RSC Under Optimized Pretreatment Conditions

According to the optimized procedure, the best conditions for phenol removal were as follows: an ethanol concentration of 84%, a ratio of 1:60 *w*/*v*, and an ultrasound duration of 15 min.

Initially, defatted RSC samples were mixed with ethanol at the specified concentration in a 1:60 ratio and stirred at 1000 rpm for 30 min on a magnetic stirrer (C-MAG HS 4, IKA, Staufen, Germany) to remove phenolic compounds. The mixture was then subjected to ultrasonic treatment in a multi-frequency ultrasonic bath (TI-H-10 2.2 gal, Elma Schmidbauer GmbH, Germany) at 45 Hz and room temperature, with occasional manual stirring using a glass rod for 15 min.

Following ultrasonication, the suspension was filtered to separate the precipitate, which was subsequently used for protein extraction as described in Section 2.2.2. The resulting protein isolate was dried and designated as Popt.

#### 2.4.4. Determination of Total Phenolic Content

The total phenolic content (TPC) in ethanolic extracts was determined using the Folin–Ciocalteu spectrophotometric method, as described by Singleton et al. [26]. To initiate the reaction, 0.1 mL of the extract was mixed with 7.9 mL of distilled water, followed by the addition of 0.5 mL of Folin–Ciocalteu’s reagent and 1.5 mL of a 20% sodium carbonate solution. The reaction mixture was then incubated in the dark at room temperature (25 °C) for 1 h. Absorbance was measured at 750 nm using a spectrophotometer (Shimadzu UV-1800, Kyoto, Japan). A calibration curve was constructed using gallic acid (0–1 mg/mL), and the results were expressed as gallic acid equivalents (mg GAE/100 g).

### 2.5. Characterization of Protein Isolates

#### 2.5.1. Sodium Dodecyl-Sulfate–Polyacrylamide Gel Electrophoresis (SDS-PAGE)

Sodium dodecyl sulfate–polyacrylamide gel electrophoresis (SDS-PAGE) is a reliable analytical method commonly employed to resolve proteins by their molecular weight. Following the protocol originally established by Laemmli [27], the protein fractions from all four concentrate samples were analyzed. The gel system was composed of two components: a 4% (*w*/*v*) acrylamide stacking layer to concentrate the samples, and a 10% (*w*/*v*) acrylamide resolving layer to achieve protein separation. Protein solutions (1 mg/mL) were prepared in Tris-Glycine buffer (pH 6.8) supplemented with 2-mercaptoethanol (50 g/L) and SDS (20 g/L). Electrophoresis was conducted using the Multi Drive XL system (Pharmacia, Uppsala, Sweden), applying 20 mA for the stacking phase and 40 mA for the resolving phase, both at 25 °C, until the tracking dye migrated to the bottom of the gel. After separation, gels were immersed in 0.2% (*v*/*v*) Coomassie Brilliant Blue R-250 staining solution prepared in 10% (*v*/*v*) acetic acid and 50% (*v*/*v*) methanol for 30 min. Excess dye was removed during a 48 h destaining process using a solution of 10% acetic acid and 40% methanol. A molecular weight marker (SDS6H2 Sigma MW Marker, Sigma-Aldrich), covering the range of 30–200 kDa, was used for molecular weight estimation of protein bands.

#### 2.5.2. Fourier-Transform Infrared Spectrum (FTIR)

The FTIR analysis was recorded at room temperature on a Nicolet iS10 Fourier transform infrared. The protein powder was pressed into a 1–2 mm slice. All spectra were taken in the spectral range of 4000–500 cm^−1^ with a 4.0 cm^−1^ resolution. The software Omnic 8.1 (Thermo Fisher Scientific, Waltham, MA, USA) was used to operate the FTIR spectrometer and collect all the data.

#### 2.5.3. Determination of Amino Acid Composition

The amino acid composition of the samples was determined using ion-exchange chromatography. Sample preparation first involved hydrolysis in 6M HCl with 0.5% phenol at 110 °C for 24 h. After hydrolysis, the samples were cooled to room temperature and dissolved in 10 mL of loading buffer (pH 2.2) (Biochrom, Cambridge, UK). The samples were filtered using 0.22 μm PTFE filters (Plano, TX, USA) and transferred into vials (Agilent Technologies, Santa Clara, CA, USA).

Amino acid analysis was performed using the Biochrom 30 Plus Amino Acid Analyzer (Biochrom, Cambridge, UK) with EZChrom Elite software version 3.3.2 (Agilent Technologies, Inc., Santa Clara, CA, USA). Amino acid detection was carried out photometrically using a UV detector at 570 nm (for all amino acids) and 440 nm (for proline). Identification was based on comparing the retention times of amino acids in the standards and samples. Quantification was performed by measuring the peak areas of amino acids and comparing them to calibration curves obtained from the standard solution (Amino Acid Standard Solution, Sigma-Aldrich, St. Louis, MO, USA). All results were expressed as grams of amino acids per 100 g of obtained protein.

#### 2.5.4. Determination of Color

The color of protein isolates was analyzed using a Minolta Chromameter (CR-400, Minolta Co., Osaka, Japan). Samples were placed in 20 mm containers and measured against black and white backgrounds at 23 ± 1 °C. Color was assessed in the CIE Lab* system, where L* represents lightness (0–100), a* the red-green axis, and b* the yellow-blue axis (both ranging from −120 to +120). Samples were mixed before each reading, and measurements were taken in triplicate.

#### 2.5.5. Determination of Protein Solubility

Protein solubility was evaluated across a pH range of 2 to 10, following a modified approach based on Popović et al. [28]. For sample preparation, 10 mg of protein concentrate was placed into Eppendorf tubes and mixed with 1 mL of appropriate buffer solution. The buffers used included HCl-KCl buffer (0.1 mol/L) for pH 2, citrate phosphate buffer (0.1 mol/L) for pH 3 and 4, phosphate buffer (0.1 mol/L) for pH 5–8, and glycine buffer (0.1 mol/L) for pH 9 and 10. Samples were incubated at 25 °C for 1 h with continuous agitation at 900 rpm using a Thermo Shaker TS-100C (BioSan, Riga, Latvia). After incubation, the samples were centrifuged at 14,500 rpm for 10 min using an Eppendorf MiniSpin Plus centrifuge (Eppendorf AG, Hamburg, Germany). The supernatants were carefully collected, and the concentration of soluble proteins was determined using the Bradford method, based on absorbance values measured at 595 nm [29].

Protein concentration was calculated according to the following equation:

(5) $$c\;\left(\frac{mg}{mL}\right)=\frac{Abs\;\times \;1.9899\;\times \;dilution\;factor}{V}$$

where

- *Abs* is the measured absorbance at 595 nm;
- *1.9899* is the calculated factor based on the BSA calibration curve;
- *dilution factor* accounts for any pre-measurement dilutions;
- *V* is the aliquot volume (mL) used in the assay.

The calibration curve was constructed using bovine serum albumin (BSA, Sigma-Aldrich, St. Louis, MO, USA), and the results were expressed as mg/mL of soluble protein.

### 2.6. Digestibility of Proteins

The in vitro digestion of all protein isolates was performed following a modified INFOGEST protocol [30]. The process involved three sequential phases, during which the mixture was continuously stirred using a magnetic stirrer in a 37 °C water bath (Velp Scientifica, Usmate Velate, Italy).

To initiate digestion, 0.5 g of protein isolate was suspended in 5 mL of distilled water and combined with 4 mL of simulated salivary fluid (SSF) electrolyte stock solution. Then, 0.5 mL of α-amylase was added, along with 25 μL of 0.3 M CaCl_2_ and 975 μL of distilled water. The pH was adjusted to 7.0 using 1 M HCl or 1 M NaOH, and the mixture was incubated at 37 °C for 2 min to simulate oral digestion.

For gastric digestion, 8 mL of simulated gastric fluid (SGF) electrolyte stock solution was added to the chyme, along with 0.04 g of pepsin, 5 μL of 0.3 M CaCl_2_, and 1.995 mL of distilled water. The pH was adjusted to 3.0 with 1 M HCl, and the sample was incubated at 37 °C for 120 min.

The final phase involved the addition of 11 mL of simulated intestinal fluid (SIF) electrolyte stock solution, 0.04 g of pancreatin, 40 μL of 0.3 M CaCl_2_, and 8.960 mL of distilled water. The pH was brought to 7.0 using 1 M NaOH, and the mixture was incubated for another 120 min at 37 °C to simulate intestinal digestion.

Simultaneously, a control sample, which underwent the same conditions without the addition of enzymes, was placed alongside the enzyme-treated sample. After digestion, the samples were centrifuged at 10,000 rpm for 10 min. The supernatant was carefully collected and stored frozen until further analysis. Digestion was assessed through the quantification of TCA-soluble proteins after digestion using the method described by Popović et al. [28], based on the quantification of soluble proteins in a 0.44 mol/L trichloroacetic acid (TCA) solution. An equal volume of cold 0.44 mol/L TCA solution was added to the protein samples, which were then incubated in a refrigerator at +4 °C for 30 min.

After incubation, the samples were centrifuged (Eppendorf MiniSpin Plus, 14,500 rpm, 10 min) to separate the insoluble proteins, while the soluble proteins remained in the supernatant. The concentration of soluble proteins in the supernatant was determined using the Lowry method [31]. Protein digestibility was assessed by comparing the concentration of TCA-soluble proteins between the control and enzyme-treated samples, with results expressed in mg/mL.

### 2.7. Statistical Analysis

Dephenolization was optimized using an artificial neural network (ANN) and particle swarm optimization (PSO) algorithm. The ANN was developed and implemented in MATLAB to predict polyphenolic yield, while the PSO algorithm, also implemented in MATLAB, was used to optimize extraction parameters.

The average values were analyzed using one-way ANOVA. Differences among treatment means were assessed using Tukey’s test at a significance level of *p* < 0.05. Statistical analysis was performed using STATISTICA software, version 13.1 (TIBCO Software Inc., Hillview Avenue, Palo Alto, CA, USA). Results are presented as mean ± standard deviation.

## 3. Results and Discussion

### 3.1. Effect of pH on the Extraction Yield and Protein Content

**Figure 1:**
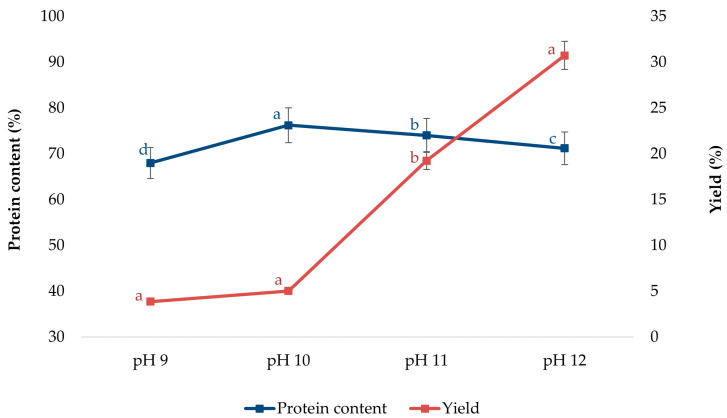
Effect of pH on protein content and yield. Values represent means of three independent determinations ± standard deviation. Different letters next to the data points indicate statistically significant differences at *p* ≤ 0.05.

Choosing the right pH value for the protein extraction from RSC can significantly affect the efficiency of the process itself. Some authors [32] revealed that raising pH values significantly enhanced the protein extraction yield, indicating that increasing the concentration of the alkaline medium might improve rapeseed protein extractability. The change in pH value is reflected in the protein solubility and, therefore, can have an impact on the protein yield during extraction. The protein yield increases with an increase in protein solubility under an alkaline medium. Hence, four different pH values (pH 9.0, 10.0, 11.0, and 12.0) were chosen for alkaline extraction from RSC. The protein content and extraction yield are presented in Figure 1.

They also reported the most favorable operational conditions for obtaining rapeseed proteins with optimal functionality and bioavailability, extracting at a pH of 9.0, followed by precipitation at pH 4.5, resulting in structurally intact proteins with reduced antinutritional compounds [8]. Also, some authors found, for the mustard meal protein extraction, that the highest yield could be obtained at pH 11, which indicated that a higher protein yield could be achieved with the increase in pH due to increased protein solubility under an alkaline medium [33]. In the literature data related to amaranth proteins, the authors also investigated the effect of pH on protein yield. They extracted proteins at pH 9, 10, 11, and 12, and obtained the highest yield at pH 12, showing that the yield increases more towards alkaline pH due to increased solubility. On the other hand, the purity of the extracted amaranth protein decreased with the increase in pH values. The best purity was obtained at pH 9. This decrease in purity as pH rises can be associated with the elevation of nonprotein nitrogen content when the pH is raised beyond the extraction pH of 9 [34].

### 3.2. Effects of Different Extraction Methods on the Extraction Yield and Protein Content

**Figure 2:**
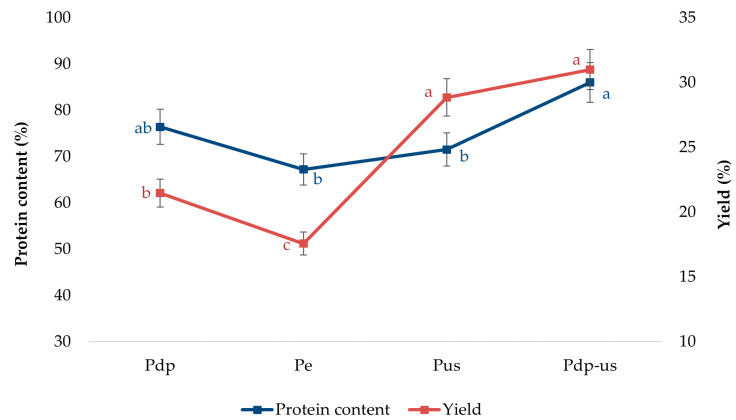
Impact of different pretreatments on protein content and yield. Values represent means of three independent determinations ± standard deviation. Different letters next to the data points indicate statistically significant differences at *p* ≤ 0.05.

After determining the optimal pH value (pH 12), for alkaline extraction, the next step in the protein extraction process from rapeseed cake was the application of appropriate pretreatments to further optimize the protein extraction protocol. The effect of four different pretreatment methods on the protein content and extraction yield of protein products is shown in the Figure 2. The protein content in the four different products followed the order Pdp-us > Pdp > Pus > Pe (Figure 2). No significant differences in protein content were observed between Pdp and Pus (*p* > 0.05), with values of 76.41% and 71.52%, respectively. The highest protein content was achieved with the Pdp-us method, which involved a combination of 80% ethanol washing and ultrasound treatment, resulting in a protein content of 86%. This represents a 12.6% increase in protein content compared to Pdp.

The protein extraction yields ranged from 17.57% to 30.99%, with the following order of yields: Pdp-us > Pus > Pdp > Pe (Figure 2). The Pdp-us method resulted in the highest extraction yield, with a 44.5% increase in yield compared to Pdp. These results suggest that ultrasonic treatment combined with ethanol washing is highly efficient in releasing bound proteins from the protein–phenol complexes, allowing for faster extraction compared to conventional alkaline extraction. The combination of alkaline extraction and physical processing techniques, such as ultrasound and ethanol washing, provides a promising and cost-effective approach that significantly increases extraction efficiency and enables scalability of the process.

Increasing the pH during extraction may contribute to higher protein yields, but it also creates the risk of unwanted reactions such as hydrolysis, theoxidation of phenolic compounds, and protein cross-linking [5]. The applied pretreatments, such as ethanol washing, were investigated with the aim of increasing protein content and reducing the presence of antinutritional factors. Specifically, hydroalcoholic extraction showed significant potential for increasing the protein content by 13% [13,14].

Ultrasonic-assisted extraction also demonstrated exceptional efficiency, increasing the protein yield by 24.6% compared to traditional methods, due to mechanisms such as cell wall disruption and enhanced protein diffusion [16]. Similar observations for rapeseed proteins, as well as for protein isolation from other plant materials, were made by other authors [35,36,37]. They highlighted the effectiveness of ultrasonic extraction as a method for releasing protein products.

In line with these results, the next step in the study was the optimization of conditions for dephenolization, aiming to remove phenolic compounds from the raw RSC. This process is crucial not only for enhancing protein availability during their isolation and reducing inhibition caused by phenolic compounds. Furthermore, optimization for scale-up could achieve increased efficiency, stability, and production capacity, while reducing costs and maintaining quality. This study focused on optimizing the process of removing phenolic compounds to improve the efficiency of protein extraction, as these compounds interfere with the extraction process and can negatively affect the quality of the obtained proteins. While phenolic compounds can contribute to nutritional value, their presence complicates protein isolation by negatively impacting yield, solubility, and structure. They also contribute to pronounced bitterness and dark coloration, which limits the use of the protein in the food industry. The amount of phenols removed in the extract was used as a key parameter for evaluating the success of the optimization [38,39,40]. After testing several treatments and pretreatments, it was determined that dephenolization had the greatest impact on protein purity, yielding a more acceptable protein. When combined with ultrasound, protein yield was also increased, further enhancing process efficiency. Thus, the removal of phenolic compounds was optimized to make the final protein more suitable for application in food products. However, these processes presented challenges, including potential protein loss, making careful optimization essential to ensure effective detoxification while preserving the highest possible protein yield. Additionally, optimizing the process is critical for achieving cost-effective and scalable production, ensuring its viability for industrial applications.

### 3.3. Impact of Process Parameters on RSC Dephenolization

**Figure 3:**
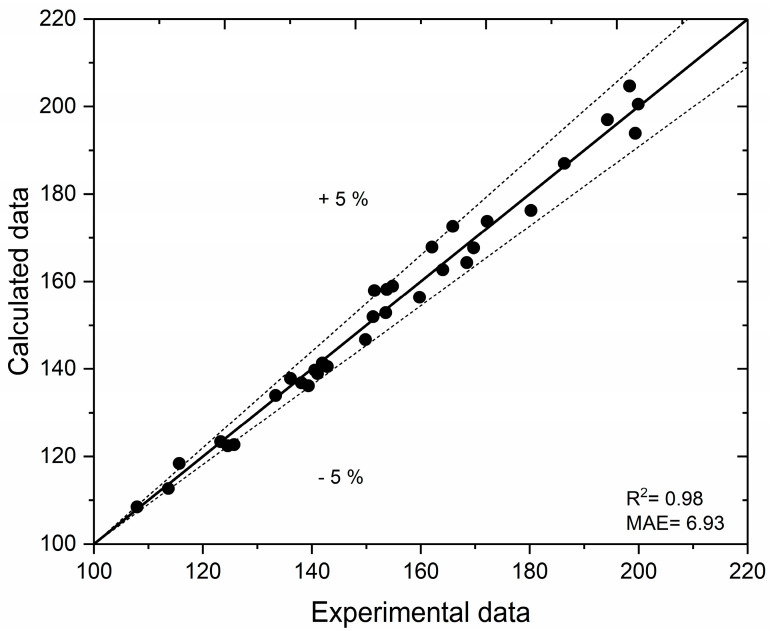
Parity plot of the predicted values of the extraction polyphenolic yield. R^2^—Coefficient of Determination; MAE—Mean Absolute Error.

A model based on artificial neural networks with predefined input parameters was employed in the optimization process. The ANN inputs were set within the following ranges: 70 < EtOH < 90%, 10 < S/L < 60 mL, and 5 < t < 25 min. In view of the limited size of the experimental dataset, adequate modeling was achieved by employing a simple ANN with a single hidden layer without inducing overfitting, according to Haykin [41]. The impact of hidden neurons on the mean R^2^ value, derived from 10 repeated training runs (with 10 initial values for each neuron), indicated that the mean R^2^ between the networks did not significantly decrease after reaching specific ANN topologies, with the mean value consistently remaining above 0.9. Consequently, the optimal architecture for the ANN included 10 neurons in the hidden layer. Figure 3 illustrates the parity plot of the experimental and predicted polyphenolic yield using the ANN with the best performance. The performance analysis of the ANN indicated good generalization of the developed neural network (R^2^ = 0.98|MAE = 6.93), enabling its use as a fitting model in subsequent approaches.

**Figure 4:**
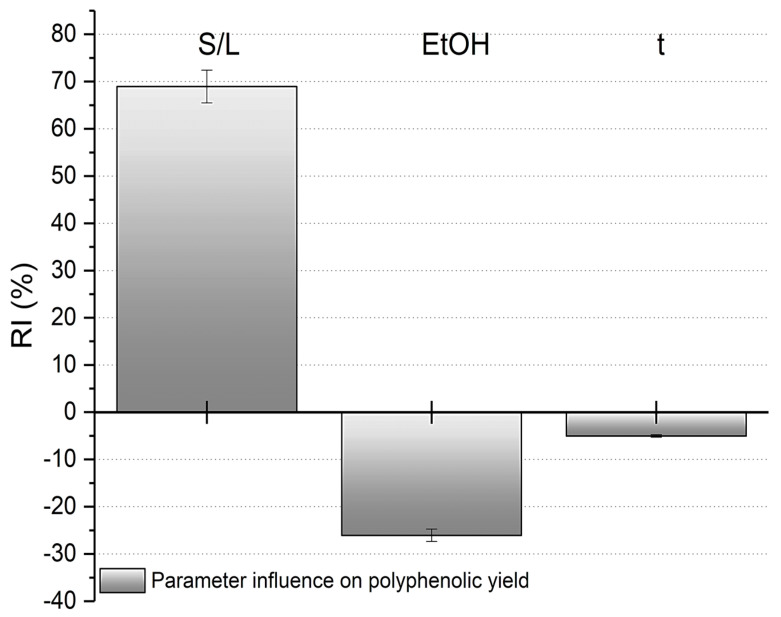
Relative importance determined using Yoon interpretation method for polyphenolic yield.

The successful creation of the ANN generated weight matrices that were used to determine the relative importance of the operating parameters and their effects (positive or negative) on polyphenolic yield (see Equation (1)). Figure 4 shows that the solid-to-liquid ratio had the greatest influence, accounting for 68.95% of the polyphenolic yield. The ethanol concentration was the second most influential parameter (26.03%), while time had a minor effect of 5.02%. The negative effect of these parameters on the polyphenolic yield indicated that as they increased, the polyphenolic yield decreased—an undesirable outcome that conflicts with the goal of maximizing phenol extraction. Based on Figure 4, it can be inferred that the extraction process is economically feasible since time had a minor influence on the yield, reducing both the time and energy consumption of the experiments.

To maximize polyphenolic yield, the particle swarm optimization (PSO) algorithm was employed. The PSO parameters in MATLAB were set as follows: 20 particles, InitialSwarmSpan = 2000, 800 iterations, equalized InertiaRange, personal learning coefficient c1 = 1.5, and global learning coefficient c2 = 2. The final operating parameters determined by the PSO were 83.3% ethanol concentration, 60% solid-to-liquid phase ratio, and 14.2 min residence time, yielding a polyphenolic content of 1887.76 mg/100 g. The PSO algorithm was run multiple times, with no variation exceeding 5% in the final results, demonstrating a strong correlation with the ANN model. These optimal parameters were successfully confirmed experimentally.

The application of optimized conditions (84% ethanol, 1:55 solid-to-liquid phase ratio, 14 min of ultrasonic treatment) and subsequent protein isolation at pH 12 with ultrasonic treatment resulted in significantly purer proteins with the best yield. The phenol content in the obtained extract was found to be 1887.76 mg/100 g, which could indicate a significant reduction in phenolic compounds in the extracted protein following the optimized defenolization process. These results are comparable to those reported by Teh et al. [23], who observed a wide range of phenolic acid contents (488.67–2104.67 mg/100 g) depending on the solvent used. The result, obtained using 84% ethanol, falls within this range and confirms the effectiveness of the selected solvent system. The pretreatment process demonstrated its effectiveness in removing both free and esterified phenolic acids, which is crucial in improving the quality of the final protein product. As a result, the protein purity reached an impressive 90.68%, with a yield of 29.17%.

In RSC, phenolic compounds exist in three forms: free, esterified, and bound [42]. The smallest proportion of phenolic acids is in the bound form, usually accounting for about 5% of the total phenolic compounds. However, sinapic acid and its derivatives, such as sinapine, which constitute approximately 70% of the free phenolic acids, are primarily responsible for the dark color and bitter taste [11,42]. Although ferulic and p-coumaric acids may contribute to the negative characteristics of the protein [11] their small proportion in RSC renders their impact negligible. These results contribute to the industrialization of rapeseed protein isolation from RSC, as improving the simple alkaline extraction process could enable the production of high-purity protein isolates.

### 3.4. Results of Protein Isolate Characterization

#### 3.4.1. Protein Profile SDS-PAGE

**Figure 5:**
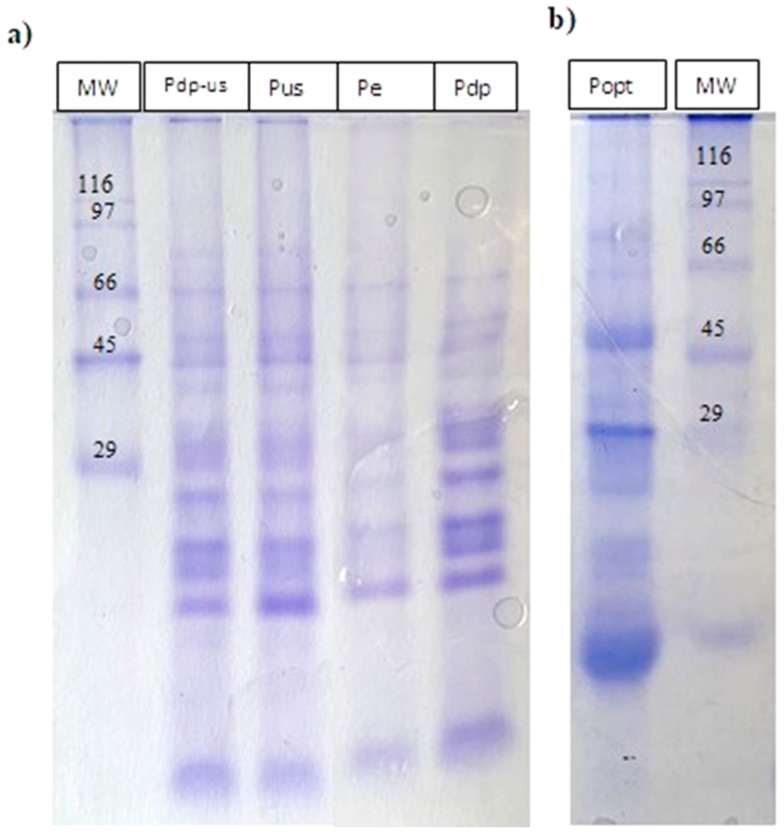
SDS-PAGE protein profiles: Pdp, Pe, Pus, and Pdp-us are shown in picture (**a**), while Popt is shown in picture (**b**). Standard protein markers are indicated by the MW line. The band below 29 kDa in the marker lane is not part of the SDS6H2 marker and was excluded from data interpretation.

SDS-PAGE gel electrophoresis was performed to analyze the protein profile of the rapeseed protein isolates. The electrophoretic profile of all obtained PIs is shown in Figure 5. The predominant proteins in rapeseed are storage proteins, napin and cruciferin, typically found in concentrations of 15–45% and 55–85%, respectively [43]. Cruciferin is a hexamer with a molecular weight of approximately 300–350 kDa. It consists of two trimers, each made up of monomers, with an α-subunit (30 kDa) linked by disulfide bonds to a β-subunit (20 kDa) [44]. Napin is a monomer with a molecular weight of approximately12–16 kDa, composed of short polypeptides of 4 kDa and long polypeptides of 9 kDa [45]. As seen in Figure 5, the most prominent bands in all samples correspond to molecular weights of 66 kDa, 45 kDa, 29 kDa, and below 29 kDa. The bands around 66 kDa and 45 kDa likely correspond to cruciferin fractions. The bands at 30.9 kDa and 28.7 kDa are associated with dissociated cruciferin α-polypeptide, while the bands at 18.8 kDa and 17.7 kDa are linked to the β-polypeptide. These findings are consistent with the study by Akbari & Wu, [46], which showed that cruciferin dissociates into α-polypeptides (26.7–37.1 kDa) and β-polypeptides (18.3–22.9 kDa). The most intense staining observed below 29 kDa may correspond to the larger polypeptides of napin [47]. This suggests that napin fractions are present in the highest concentration, as they are more soluble in water and under alkaline conditions. Protein products obtained using methods involving ethanol washing and ultrasonic treatment (Pdp, Pus, and Pdp-us) contained these same fractions, while protein isolate obtained through the enzyme-based method (Pe) primarily contained cruciferin fractions with higher molecular weights (~45 kDa). The bands at the lower end of the SDS-PAGE gel could be attributed to dissociated napin or its smaller polypeptides, which have a reported molecular weight of approximately 4.5 kDa [8,48]. A detailed understanding of the protein profile of PI is crucial, as the napin-to-cruciferin ratio in the isolate significantly affects the techno-functional properties of the proteins. This is particularly important given their distinct functional characteristics, with napin contributing to foaming properties, whereas cruciferin exhibits gelling behavior, thus influencing the potential applications of the isolated proteins [7].

#### 3.4.2. FTIR

**Figure 6:**
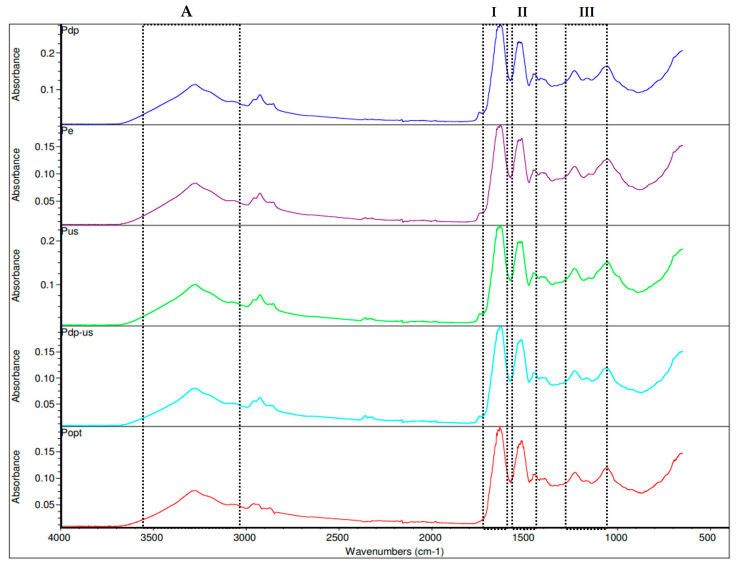
Fourier-transform infrared (FTIR) spectrum of the rapeseed protein isolates: A—amide A (3000–3750 cm^−1^), I—amide I (1700–1600 cm^−1^), II—amide II (1600–1400 cm^−1^), III—amide III (1300–1200 cm^−1^).

FT-IR spectroscopy was one of the key methods for analyzing the protein structure [49]. Generally, the infrared spectra profiles of PIs were similar (Figure 6). Spectra in the range of 400–4000 cm^−1^ were recorded to identify the functional groups in the protein isolates (Figure 6). All isolates exhibited characteristic absorption bands associated with proteins, notably in the regions of amide A (3500–3000 cm^−1^), amide I (1700–1600 cm^−1^), amide II (1550–1500 cm^−1^), and amide III (1300–1200 cm^−1^).

The most prominent peaks were observed at approximately 1633 cm^−1^ (C=O stretching, amide I band), 1532 cm^−1^ (N–H bending, amide II band), and 1232 cm^−1^ (C–N stretching and N–H bending, amide III band), consistent with typical protein spectra [50]. In addition, a broad band around 3300 cm^−1^ corresponds to O–H and N–H stretching vibrations.

Amide I and II regions are highly sensitive to protein secondary structures, particularly α-helices and β-sheets. The peak near 1633 cm^−1^ typically indicates a prevalence of α-helical structures, whereas peaks below 1630 cm^−1^ are often associated with β-sheets [51,52]. In the current study, subtle differences were noted between the spectra of the isolates. The Popt isolate, obtained through ANN-optimized dephenolization followed by ultrasound-assisted protein extraction, showed slightly sharper and more intense peaks in the amide I and II regions. This suggests a better preservation of ordered secondary structures, possibly due to milder processing conditions and the effective removal of phenolic prior to extraction. In contrast, the Pe and Pus isolates exhibited slight shifts and peak broadening in the same regions, potentially indicating partial protein unfolding or a reduction in α-helical content, as has been previously associated with conformational changes [53,54].

Additionally, the interaction between residual phenolic compounds and proteins appears to have influenced the spectral characteristics. Phenolic compounds can interact with proteins through hydrogen bonding or covalent mechanisms, particularly under oxidative conditions, leading to decreased band intensity and peak broadening in the amide regions. These effects were especially evident in the Pe and Pus samples, which likely retained higher levels of phenolic compounds [55].

#### 3.4.3. Amino Acid Composition

The amino acid profile is presented in Table 1, and all observed amino acids were detected in all samples, indicating that PIs have a beneficial amino acid composition capable of meeting human dietary needs. In fact, its profile can be compared to that of widely used proteins such as egg and milk [56].

The highest observed amino acid content (98.58%) was found in the optimal sample, Popt. This trend was consistent among essential amino acids (EAAs), with Popt (41.32%) showing the highest levels. Similarly, the highest concentrations of non-essential amino acids (NEAAs) were also found in the same sample Popt (57.26%). Among the essential amino acids, leucine, valine, and lysine showed the highest concentrations. When compared with the WHO adult requirements for essential amino acids [57] nearly all of the EAA values in the rapeseed protein samples exceeded the recommended intake levels. While some studies suggest that lysine is a limiting amino acid in RSC proteins [45,58], in this study, lysine levels in all samples were above the WHO minimum threshold of 4.5%. This may be explained by the fact that lysine is a heat-sensitive amino acid, and its content is often influenced by the thermal processing of the RSC. Since this study did not use such processing, lysine levels were preserved. Among the non-essential amino acids, the highest concentrations were found in glutamic acid, arginine, and aspartic acid. The ratios of EAA/NEAA (69.00–74.00%) and EAA/TAA (41.00–43.00%) in all PIs were higher than the FAO/WHO recommended ratios of 60% [57], suggesting that the rapeseed proteins possess an optimal amino acid profile. These results indicate that all protein extraction methods from RSC produced proteins with high total amino acid (TAA) content. However, additional processing steps, such as ethanol washing and ultrasound treatment (applied Popt), likely enhanced the amino acid profile in the final product. This aligns with the findings of Zhang et al. [59], who observed similar amino acid patterns when employing comparable extraction techniques.

#### 3.4.4. Color Analysis

Color plays a crucial role in determining the applicability of protein ingredients in the food industry [47]. Due to the presence of phenolic compounds in (RSC), protein isolates derived from it often exhibit a dark green color, which is not desirable from a sensory perspective. The intense color is a result of complexes formed by the covalent binding of phenols to proteins.

Table 2 presents the difference in color of PIs obtained through all methods of extraction. The values obtained for the L*, a*, and b* parameters were similar for all samples. The obtained values for the parameter L* were around 50 and indicated that the isolates are still quite dark in color and that complexes leading to the discoloration of the protein product are still present. It is worth noting that vacuum drying, used in this study, may contribute to the darker color of the samples due to possible Maillard reactions or the concentration of pigments. In contrast, milder drying techniques such as freeze drying (lyophilization) may result in lighter colored PIs by better preserving their native appearance.

The protein isolates obtained after the optimization of the dephenolization process exhibited lower parameter a* values, indicating a reduction in green color, and higher parameter b* values, signifying an increase in yellow color. Although the overall brightness (parameter L* value) did not increase significantly, the color shift from green to yellow suggests that the dephenolization process significantly contributes to the sensory acceptability of the final isolates for further application.

#### 3.4.5. Protein Solubility

**Figure 7:**
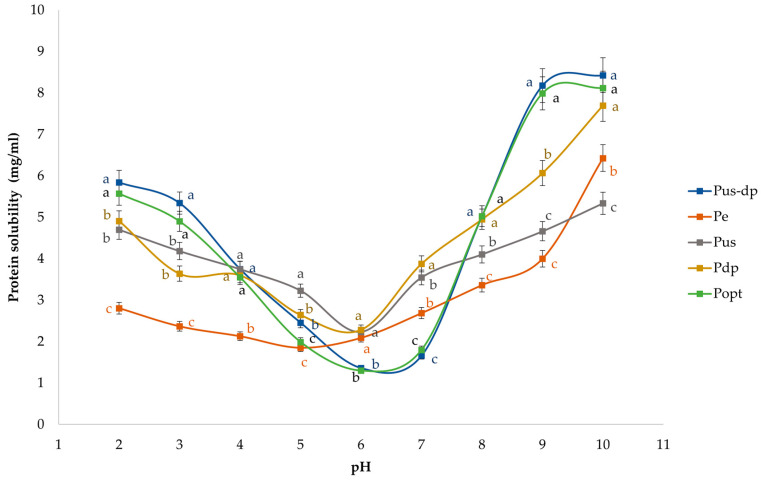
The solubility profile (pH dependent profile) of PIs obtained through different extraction methods. Values represent means of three independent determinations ± standard deviation. Different letters next to the data points indicate statistically significant differences at *p* ≤ 0.05, with each color corresponding to a specific treatment.

Protein solubility is an essential functional property that influences the application of proteins in food formulations, affecting attributes such as emulsification, foaming, and gelation. Additionally, it provides valuable insight into the structural and physicochemical characteristics of proteins, reflecting changes induced by different extraction and pretreatment methods. Therefore, the solubility of PIs obtained by different extraction pre-treatment methods was investigated and presented in Figure 7.

The obtained results exhibited a characteristic U-shaped solubility profile, consistent with previous studies on oilseed proteins [47]. The lowest solubility of all PIs varied slightly, occurring within the values of pH 5 and pH 6, suggesting minor differences in the isoelectric point (pI) of the proteins due to different extraction treatments.

The highest solubility was observed for the PI obtained through the combined ultrasound and dephenolization treatments (Pdp-us), as well as the PI obtained through optimized dephenolization conditions (Popt), particularly in the alkaline pH range (8–10). These results demonstrate that optimized extraction parameters effectively enhance protein solubility, supporting the key role of the removal of phenolic compounds in facilitating protein solvation [50]. The protein isolate obtained through dephenolization alone (Pdp) demonstrated significantly higher solubility compared to both the ultrasound-pretreated (Pus) and enzyme-pretreated (Pe) samples, further highlighting the major contributing factor of phenolic compound removal in improving protein functionality. Although ultrasound pretreatment resulted in a protein isolate (Pus) with slightly reduced solubility, it remained higher than the enzyme-pretreated sample (Pe). This indicates that ultrasound treatment, through mechanisms such as cavitation and the partial disruption of intermolecular interactions [60], enhances protein solubility, although the effectiveness of this pretreatment is significantly enhanced when accompanied with the dephenolization process.

However, the enzyme-treated sample (Pe) generally exhibited the lowest solubility across the tested pH range. This finding could be due to the limited effectiveness of cellulase treatment in breaking down the cell wall matrix, which hindered the release of soluble protein fractions and was insufficient to liberate phenolic compounds bound within the matrix. As a result, phenolics likely remained associated with the protein structures, forming insoluble complexes that reduced overall solubility [61]. However, a slight increase in solubility observed at strongly alkaline conditions (around pH 10) suggests that some proteins became more accessible for hydration under these extreme pH conditions [8].

These findings were supported by SDS-PAGE analysis. PIs exhibiting higher solubility (Popt, P dp-us, and Pdp) displayed intense bands below 29 kDa, probably associated with napin fractions due to their higher solubility. In contrast, the enzyme-pretreated isolate (Pe) had more pronounced bands around 45 kDa, corresponding to cruciferin fractions (complex quaternary structure) that show limited solubility [48].

### 3.5. Protein Digestibility Analysis

**Figure 8:**
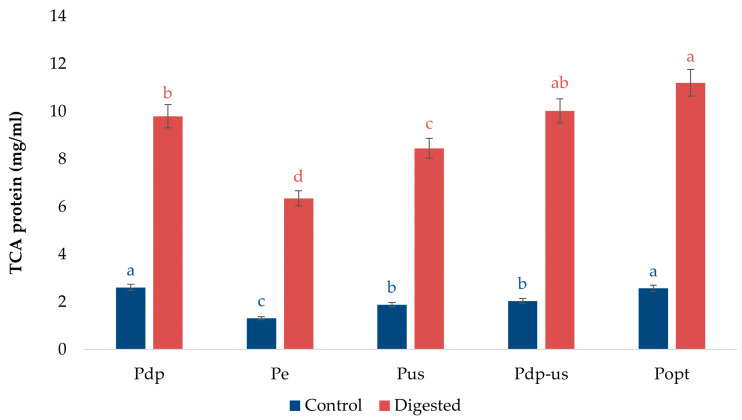
TCA-soluble protein content before and after in vitro digestion of PIs obtained using different extraction methods. Values represent means of three independent determinations ± standard deviation. Different letters next to the data points indicate statistically significant differences at *p* ≤ 0.05, with each color corresponding to a specific treatment.

In vitro digestion was conducted to assess the digestibility of the protein isolates, an important indicator of their nutritional quality and potential bioavailability. The TCA-soluble protein content of RPs provides insight into their digestibility. The results (Figure 8) show a significant increase (*p* < 0.05) in TCA-soluble protein concentration in all analyzed samples following digestion compared to the undigested controls, suggesting the efficiency of proteolysis.

Pop and Pdp-us exhibited the highest TCA-protein content after digestion, which indicated their highest digestibility among all protein isolates. This enhanced digestibility probably resulted from structural changes in the protein caused by ultrasound and the removal of phenolic compounds, which increased protein accessibility and facilitated more efficient enzymatic hydrolysis. Pdp also showed high digestibility, while the sample treated only with ultrasound (Pus) had slightly lower TCA-soluble protein content in comparison with other samples. The moderate digestibility of Pe may be attributed to the retention of larger molecular weight protein fractions that require extended hydrolysis and different proteolysis conditions.

In contrast, dephenolization pretreatment (Pdp) positively influenced digestibility, most likely by reducing the presence of phenolic compounds that can interfere with enzymatic activity by forming insoluble protein–phenol complexes. The negative impact of such complexes on protein digestibility has been previously demonstrated in studies on protein–phenol conjugates [62]. Samples subjected to ultrasound pretreatment (Pus and Pdp-us) demonstrated improved digestibility compared to the control samples, probably due to ultrasound induced structural alterations that facilitated enhanced enzyme accessibility.

The results indicate that the applied processing methods had a significant effect on rapeseed protein digestibility. Specifically, ultrasound and dephenolization pretreatments resulted in the highest concentrations of TCA-soluble proteins, reflecting a more extensive enzymatic hydrolysis. The presence of peptides after digestion suggests potential bioactivity, which is particularly interesting from a nutritional and functional perspective. Previous studies have shown that rapeseed proteins can release peptides with antihypertensive, antioxidant, and antimicrobial properties upon hydrolysis [63,64,65,66]. The peptides generated during digestion may retain such bioactivities upon consumption, while their bioavailability and bioaccessibility could be the subject of future investigations.

## 4. Conclusions

The conclusion of this study shows that optimizing protein extraction methods from rapeseed significantly reduces the content of phenolic compounds, leading to improvements in the functional and sensory properties of the obtained proteins. Removing phenolic compounds is crucial for increasing the nutritional value of proteins and their acceptability in human nutrition, thus opening new opportunities for the application of rapeseed proteins in the food industry. The comparison of different extraction methods revealed that combining dephenolization with ultrasound pretreatment enabled efficient removal of phenolic compounds, while preserving protein yield and maintaining favorable functional properties.

The results of this study highlight the importance of optimizing the extraction process by applying advanced techniques such as ultrasound treatment and modeling using ANNs. The optimal extraction conditions for the dephenolization of RSC include an ethanol concentration of 84%, a solid-to-liquid ratio of 1/60 *w*/*v*, and a 15 min ultrasound treatment. These conditions resulted in an impressive protein purity of 90.68%, with a yield of 29.17%.

Further research should focus on implementing these techniques under real industrial conditions, as well as analyzing their impact on the long-term stability and sensory properties of proteins. Additionally, further optimization of the parameters could contribute to cost reduction and improved sustainability of the process, thereby enabling the commercialization of rapeseed proteins as a valuable ingredient in food products.

## Figures and Tables

**Table 1:** Amino acid profile of the rapeseed protein isolates.

Amino Acids (g/100 g Proteins) *	Pdp	Pe	Pus	Pdp-us	Popt
Thr	4.55 ± 0.07 ^b^	5.20 ±0.04 ^a^	4.94 ± 0.08 ^a^	4.56 ± 0.07 ^b^	4.97 ± 0.11 ^a^
Val	5.14 ± 0.04 ^c^	5.27 ± 0.06 ^bc^	5.14 ± 0.08 ^c^	5.42 ± 0.08 ^b^	5.93 ± 0.06 ^a^
Met	3.10 ± 0.04 ^b^	2.58 ± 0.06 ^c^	2.58 ± 0.08 ^c^	3.02 ± 0.06 ^b^	3.35 ± 0.06 ^a^
Ile	4.31 ± 0.08 ^ab^	4.58 ± 0.07 ^a^	4.35 ± 0.04 ^ab^	4.21 ± 0.03 ^b^	4.44 ± 0.10 ^ab^
Leu	6.71 ± 0.03 ^c^	7.10 ± 0.07 ^b^	6.73 ± 0.08 ^c^	6.83 ± 0.04 ^c^	7.57 ± 0.08 ^a^
Phe	4.83 ± 0.04 ^bc^	5.14 ± 0.07 ^a^	4.96 ± 0.03 ^ab^	4.65 ± 0.04 ^c^	4.96 ± 0.07 ^ab^
Lys	6.95 ± 0.03 ^ab^	6.21 ± 0.10 ^c^	6.65 ± 0.13 ^b^	6.71 ± 0.03 ^b^	7.24 ± 0.11 ^a^
His	2.97 ± 0.04 ^a^	2.71 ± 0.06 ^bc^	2.76 ± 0.08 ^abc^	2.57 ± 0.04 ^c^	2.86 ± 0.07 ^ab^
Ʃ EAA	38.56 ± 0.01 ^bc^	38.79 ± 0.13 ^b^	38.11 ± 0.37 ^bc^	37.97 ± 0.17 ^c^	41.32 ± 0.05 ^a^↑
Asp	8.42 ± 0.03 ^c^	9.21 ± 0.11 ^ab^	8.92 ± 0.10 ^b^	9.07 ± 0.03 ^b^	9.47 ± 0.14 ^a^
Ser	4.94 ± 0.11 ^b^	5.23 ± 0.03 ^b^	5.20 ± 0.10 ^b^	5.12 ± 0.07 ^b^	5.55 ± 0.04 ^a^
Glu	13.68 ± 0.07 ^c^	13.11 ± 0.11 ^d^	13.19 ± 0.14 ^d^	15.20 ± 0.07 ^b^	16.79 ± 0.14 ^a^
Gly	4.21 ± 0.07 ^c^	4.16 ± 0.03 ^c^	4.10 ± 0.06 ^c^	4.61 ± 0.03 ^b^	5.14 ± 0.10 ^a^
Ala	4.04 ± 0.04 ^c^	4.07 ± 0.06 ^c^	4.05 ± 0.03 ^c^	4.35 ± 0.08 ^b^	5.01 ± 0.06 ^a^
Tyr	3.30 ± 0.08 ^c^	3.95 ± 0.07 ^a^	3.70 ± 0.04 ^b^	3.18 ± 0.06 ^c^	3.16 ± 0.03 ^c^
Arg	8.30 ± 0.08 ^a^	7.55 ± 0.03 ^c^	8.14 ± 0.11 ^ab^	7.90± 0.03 ^b^	6.32 ± 0.04 ^d^
Pro	6.16 ± 0.03 ^a^	5.05 ± 0.03 ^e^	5.38 ± 0.04 ^d^	5.61 ± 0.07 ^c^	5.82 ± 0.06 ^b^
Ʃ NEAA	53.05 ± 0.13 ^c^	52.33 ± 0.07 ^c^	52.68 ± 0.14 ^c^	55.04 ± 0.04 ^b^	57.26 ± 0.35 ^a^
Ʃ TAA	91.61 ± 0.11 ^c^	91.12 ± 0.20 ^c^	90.79 ± 0.51 ^c^	93.01 ± 0.21 ^b^	98.58 ± 0.40 ^a^↑
EAA/NEAA (%)	73.00	74.00	72.00	69.00	72.00
EAA/TAA (%)	42.00	43.00	42.00	41.00	42.00

* on dry basis; NEAA—non-essential amino acids; EAA—essential amino acids; TAA—total amino acids. Values represent means of two independent determinations ± standard deviation. Different letters next to the data points indicate statistically significant differences at *p* ≤ 0.05. The symbol ‘↑’ represents a significant increase.

**Table 2:** Color parameters of the rapeseed protein isolates.

Samples	Pdp	Pe	Pus	Pdp-us	Popt
L*	50.34 ± 0.1 ^b^	49.80 ± 0.30 ^b^	51.88 ± 0.18 ^a^	47.78 ± 0.26 ^c^	31.46 ± 0.15 ^d^
a*	6.02 ± 0.05 ^a^	5.08 ± 0.07 ^c^	5.21 ± 0.11 ^c^	5.83 ± 0.04 ^b^	3.43 ± 0.05 ^d^
b*	18.10 ± 0.22 ^b^	17.20 ± 0.17 ^c^	20.52 ± 0.19 ^a^	17.86 ± 0.09 ^b^	7.45 ± 0.08 ^d^

Values represent means of three independent determinations ± standard deviation. Different letters next to the data points indicate statistically significant differences at *p* ≤ 0.05.

## Data Availability

The original contributions presented in the study are included in the article/Supplementary Material; further inquiries can be directed to the corresponding author.

## Supplementary Materials

The following supporting information can be downloaded at: https://www.mdpi.com/article/10.3390/foods14101762/s1, Table S1: Phenolic compound removal from RSC: 33-step experimental design and two optimized procedures based on ANN modelling.
