# Supplementary material for: Optimization of Protein Extraction from Rapeseed Oil Cake by Dephenolization Process for Scale-Up Application Using Artificial Neural Networks
Source: Foods. 2025 May 7;14(10):1762. doi: 10.3390/foods14101762 (PMC12111404; doi:10.3390/foods14101762)
Supplement: Supplementary file 1 [file foods-14-01762-s001.zip › foods-3599362-supplementary.pdf]

Table S1. Phenolic compound removal from RSC: 33-step experimental design and two optimized procedures based on ANN modelling

Sample	t (min)	Ratio	EtOH	TPC (mg GEA/100g)
**2**	25	20	80	1587.47
**7**	25	10	80	1318.20
**21**	25	15	80	1479.24
**3**	15	10	80	1297.20
**9**	15	20	80	1482.68
**15**	15	15	80	1420.28
**5**	5	15	80	1430.11
**14**	5	20	80	1433.18
**17**	5	10	80	1333.85
**1**	25	15	70	1577.34
**13**	25	20	70	1610.45
**27**	25	10	70	1394.72
**11**	15	10	70	1291.64
**18**	15	20	70	1549.90
**19**	15	15	70	1489.46
**12**	5	20	70	1516.46
**16**	5	10	70	1326.56
**23**	5	15	70	1482.16
**6**	25	20	90	1289.71
**22**	25	10	90	1164.87
**25**	25	15	90	1242.17
**8**	15	20	90	1254.80
**20**	15	10	90	1059.05
**26**	15	15	90	1128.92
**4**	5	15	90	1089.17
**10**	5	20	90	1105.77
**24**	5	10	90	1017.47
**28**	25	25	70	1608.36
**29**	25	30	70	1686.79
**30**	25	40	70	1734.24
**31**	25	50	70	1761.14
**32**	25	55	70	1777.33
**33**	25	60	70	1801.54
**OPT1**	25	60	71	1830.28
**OPT2**	25	60	84	1887.76
